# Electrochemical Detection of Hydrogen Peroxide by Inhibiting the *p*-Benzenediboronic Acid-Triggered Assembly of Citrate-Capped Au/Ag Nanoparticles on Electrode Surface

**DOI:** 10.3390/ma10010040

**Published:** 2017-01-05

**Authors:** Lin Liu, Ting Sun, Huizhu Ren

**Affiliations:** Henan Province of Key Laboratory of New Optoelectronic Functional Materials, College of Chemistry and Chemical Engineering, Anyang Normal University, Anyang 455000, China; sunting@aynu.edu.cn (T.S.); ren_huizhu@sohu.com (H.R.)

**Keywords:** boronic acid, hydrogen-peroxide, metal nanoparticles, electrochemical sensors, colorimetric assay

## Abstract

Metal nanoparticles (NPs) possess unique physicochemical attributes for creating effective recognition and transduction processes in chem/bio-sensing. In this work, we suggested that citrate-capped Au/Ag NPs could be used as the reporters for the design of hydrogen peroxide (H_2_O_2_) sensors with a simple manipulation principle and an easy detection procedure. Specifically, *p*-benzenediboronic acid (BDBA) induced the aggregation of citrate-capped Au NPs through the cross-linking reaction between citrate and boronic acid of BDBA in solution. By modifying the electrode with a boronic acid derivative, the BDBA-induced assembly of Au NPs was achieved on the electrode surface. This led to a significant decrease in the electron transfer resistance due to the unique conductive ability of Au NPs. However, when the boronate group on the electrode surface was oxidized into its phenol format, the assembly of Au NPs on the electrode surface was not achieved. As a result, a higher electron transfer resistance was observed. The process could be monitored by electrochemical impedance technique. Furthermore, when Ag NPs were used instead of Au NPs in this design, the H_2_O_2_ concentration could be determined by measuring the linear-sweep voltammetry (LSV) current through the solid-state Ag/AgCl reaction of Ag NPs. The results indicated that NP-based colorimetric assays could be developed into more sensitive electrochemical analysis.

## 1. Introduction

Simple, cost-effective and highly sensitive chemical and biological sensors feature two functional components. One is the recognition element providing a specific interaction with the target analyte. The other is the transducer component for the sensor signal output. Metal (Au/Ag in particular) nanoparticles (NPs) possess unique physicochemical attributes which have facilitated them in being used for creating effective recognition and transduction processes in chem/bio-sensing in the last decades [1,2,3]. In contrast to other materials for the design of sensors, Au/Ag NPs offer clear advantages, such as a simple preparation procedure, a size-dependent optical property, facile surface modification, excellent conductivity, and high surface area and/or good catalytic ability [4,5,6]. The Au/Ag NPs–based sensing techniques usually include colorimetry, fluorescence, electrochemistry, localized surface plasmon resonance, surface enhanced Raman scattering (SERS), quartz crystal microbalance, and bio-barcode assay [7,8,9,10,11,12,13,14]. Among these sensing strategies, colorimetric assays based on target recognition–induced aggregation or redispersion of Au/Ag NPs in particular have been prevalent recently because of their simple manipulation principle and easy detection procedure [8,12]. However, most colorimetric assays show low sensitivity. Thus, the colorimetric examples were expected to re-create existing platforms with improved sensitivity. By simply incorporating the colorimetric principle or technique into another field, new achievements were produced recently [14,15,16,17,18]. For example, based on the high quenching efficiency of dispersed but not aggregated Au NPs on the fluorescence of organic dyes or quantum dots (denoted as the efficient inner filter effect), visual and fluorescent sensors could be developed [14,15,16]. Furthermore, Wei et al. first suggested that the target-induced aggregation of Ag NPs in solution could be facilely initiated on the solid-liquid surface, which thus converted a liquid-phase Hg^2+^ colorimetric assay into an electrochemical analysis with improved sensitivity [18].

Hydrogen peroxide (H_2_O_2_) plays an important role in the immune system and functions as a signaling molecule in the regulation of a wide variety of biological processes [19,20]. The concentration change of H_2_O_2_ has been demonstrated to be closely correlated with many diseases, such as chronic inflammation, diabetes, neurodegenerative disorders and cancers [21,22]. Moreover, H_2_O_2_ is an enzymatic product used in the laboratory as an indicator to measure the target concentration [23,24]. Thus, both in vivo and in vitro determination of H_2_O_2_ is of great significance. The most common techniques used for H_2_O_2_ detection at present are fluorescence and electrochemistry which employ fluorescent probes and enzyme-/nanomaterial-modified electrodes, respectively. We noticed that a few of the fluorescent H_2_O_2_ sensors are based on the selective and efficient reaction between boronate groups (either boronic acid or boronate ester) and H_2_O_2_ [25,26,27,28,29,30]. The boronate group in a fluorescent probe is usually removed by H_2_O_2_ to yield its corresponding phenol form, thus resulting in an increase/decrease of the fluorescence intensity or a shift of the fluorescence peak. More interestingly, we also noticed that boronic acid can react with citrate to form a boronic acid–citrate complex and the citrate-capped Au/Ag NPs could be simply prepared [31,32,33,34]. Inspired by these facts, herein we conceived a simple electrochemical strategy for the sensitive and selective detection of H_2_O_2_ by converting a liquid-phase colorimetric assay into an electrochemical analysis. In the colorimetric assay, *p*-benzenediboronic acid (BDBA) induced the assembly of citrate-capped Au NPs through the formation of boronate ester in solution. Once BDBA was oxidized by H_2_O_2_ into the phenol form, it would lose the ability to trigger the assembly of Au NPs. Since the gold electrode exhibits a superficial microenvironment similar to that of Au/Ag NPs, the BDBA-induced assembly of Au NPs can be facilely initiated on the boronic acid–covered electrode surface through the formation of boronate ester. However, removing the boronate group by H_2_O_2_ either on the electrode surface or in BDBA would prevent the assembly of Au NPs. Since Au NPs show excellent electrical conductivity, this process could be easily monitored by electrochemical impedance spectroscopy (EIS, a powerful electrochemical technique for studying the surface process and properties) [35,36,37]. When Ag NPs were used instead of Au NPs, the signal could be measured by linear-sweep voltammetry (LSV) through the solid-state Ag/AgCl reaction of Ag NPs [18]. The electrochemical strategy not only features a simple manipulation principle and an easy detection procedure similar to that of the colorimetric assay but also shows high sensitivity and specificity.

## 2. Results and Discussion

### 2.1. Colorimetric Assay of H_2_O_2_

**Figure 1:**
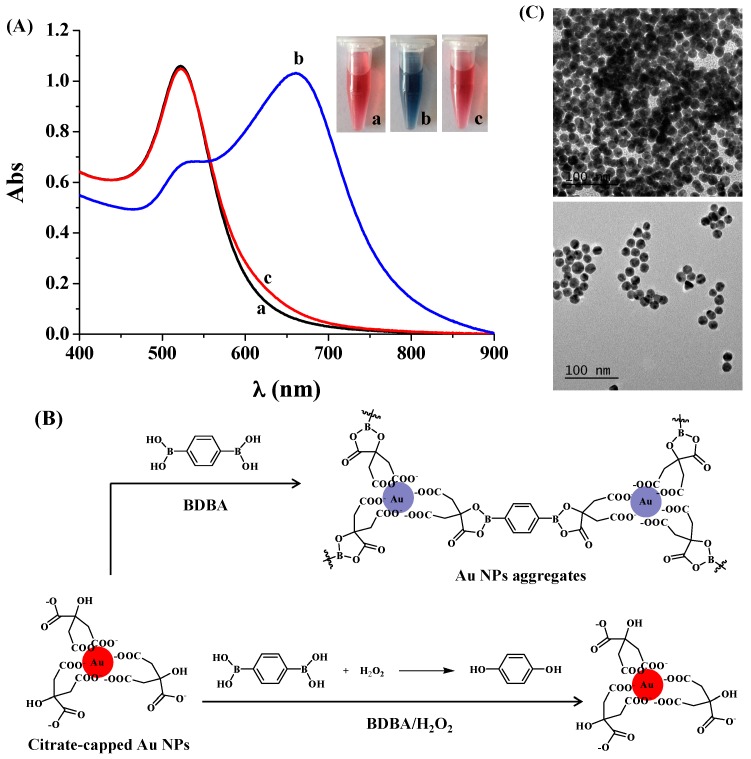
(**A**) UV/vis absorption spectra and optical photographs of 4 nM Au NPs in the absence (curve/tube a) and presence of BDBA (curve/tube b) or H_2_O_2_-treated BDBA (curve/tube c). The final concentrations of BDBA and H_2_O_2_ used were 0.6 mM and 1 mM, respectively; (**B**) Schematic illustration of BDBA-induced aggregation of citrate-capped Au NPs and reaction between BDBA and H_2_O_2_; (**C**) TEM images of Au NP suspension in the presence of BDBA (top) and H_2_O_2_-treated BDBA (bottom).

**Figure 2:**
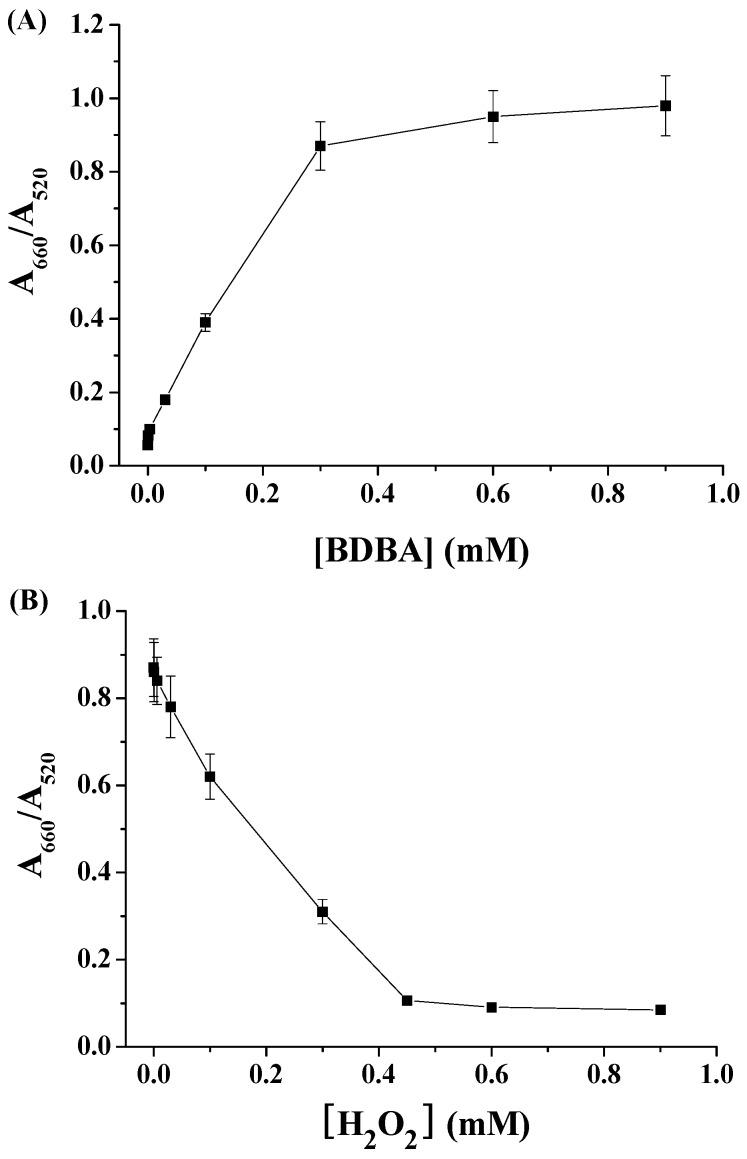
(**A**) Effect of BDBA concentration on the A_660_/A_520_ ratio of 4 nM Au NPs; (**B**) Dependence of A_660_/A_520_ on the concentration of H_2_O_2_. The final concentration of used BDBA was 0.3 mM.

To demonstrate that BDBA could induce the aggregation of citrate-capped Au NPs and that H_2_O_2_ could inhibit the BDBA-triggered NP aggregation, we first investigated the color and UV/vis absorption change of the Au NP suspension in the presence of BDBA/H_2_O_2_. As shown in Figure 1A, compared to the extinction spectrum of citrate-capped Au NPs, the addition of BDBA caused the color change of the Au NP suspension from red to blue, which was accompanied by a decrease in the absorption at 520 nm and the appearance of a new absorption peak at ~660 nm. The red-shift band of the UV/vis spectra and the red-to-blue color change are characteristic of Au NP aggregation. The aggregation should contribute to the covalent interaction between boronate in BDBA and citrate on the surface of Au NPs (Figure 1B) [31,32,33,34]. However, the mixture of BDBA/H_2_O_2_ did not cause significant changes in the color and absorption spectrum of Au NPs. This suggested that the H_2_O_2_-treated BDBA did not induce Au NP aggregation, which was further confirmed by the TEM observation (Figure 1C): there were aggregated Au NPs in the presence of BDBA and dispersed Au NPs in the presence of the BDBA/H_2_O_2_ mixture. The result can be attributed to the fact that H_2_O_2_ can react with boronate-derived group to form its phenol form [25,26,27,28,29,30]. We further investigated the influence of both BDBA and H_2_O_2_ concentrations on the absorption change of Au NPs. The A_660_/A_520_ ratio (wherein A_660_ and A_520_ represent the absorption intensity of Au NPs at 660 nm and 520 nm, respectively) increased linearly in the concentration range of 3–300 µM (Figure 2A). In contrast, it decreased with an increased H_2_O_2_ concentration ranging from 6 to 450 µM (Figure 2B).

### 2.2. Principle of Electrochemical Assay of H_2_O_2_ by Au NPs

**Figure 3:**
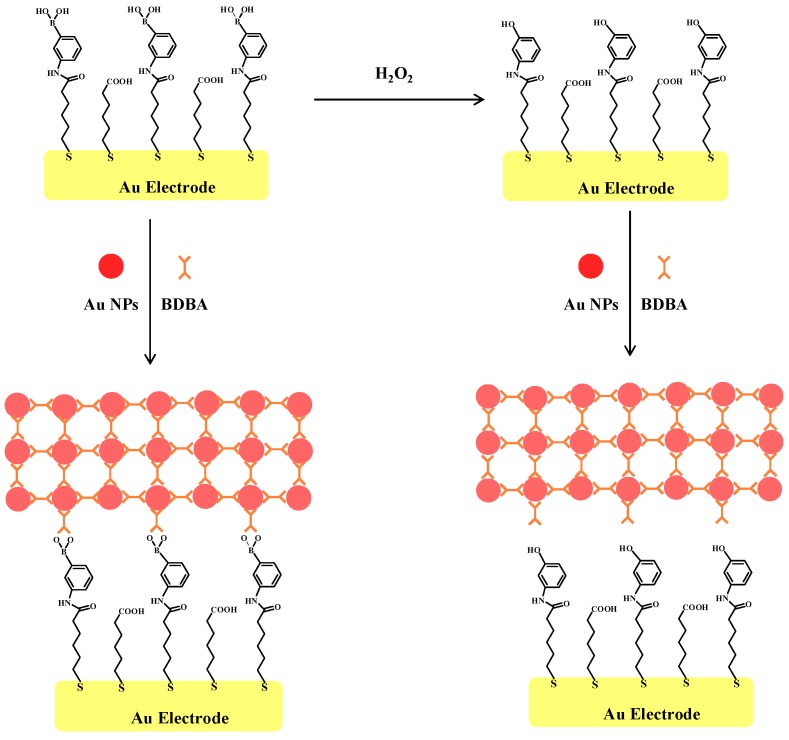
Illustration of the electrochemical strategy for H_2_O_2_ based on BDBA-induced assembly of citrate-capped Au NPs on boronate-covered gold electrode surface.

The principle of the Au NP–based electrochemical method is shown in Figure 3. The boronate self-assembled monolayer (SAM) behaves as a barrier for [Fe(CN)_6_]^3−/4−^. According to the design, the boronate-covered gold electrode can capture citrate-capped Au NPs and BDBA molecules in solution through the formation of boronate ester bonds. Surface-tethered Au NPs and BDBA can recruit more Au NPs and peptides, thus leading to the formation of a network of NPs-BDBA-NPs-BDBA on the electrode surface. The unique conductive ability of Au NPs may result in a significant change in the charge transfer resistance. However, once the electrode was incubated with H_2_O_2_, boronate groups on the electrode surface were removed. As a result, the citrate-capped Au NPs could not be captured by the electrode. Note that citrate-capped Au NPs maybe absorb other components in a biological sample and boronate can react with diol-containing biomolecules (e.g., sugar, catechol derivatives and glycoproteins) [38,39,40]; thus, the detection of H_2_O_2_ was performed by incubating the electrode with the H_2_O_2_ sample before introducing Au NPs and BDBA on the electrode surface. Although boronic acid can covalently react with diol-containing biomolecules to form five- or six-membered cyclic ester in an alkaline aqueous solution, the cyclic ester can dissociate when the medium is changed to acidic pH. Thus, the captured diol-containing biomolecules could be detached by rinsing the electrode with 10 mM HCl after the step of treatment by the H_2_O_2_ sample, thus avoiding the interference of diol-containing biomolecules and facilitating the formation of a network of citrate-capped Au NPs.

### 2.3. Electrochemical Detection of H_2_O_2_ Based on the Signal Amplification of Au NPs

**Figure 4:**
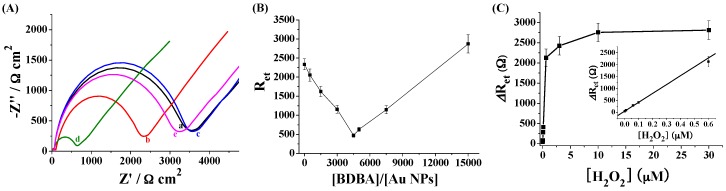
(**A**) EIS of the boronate-covered electrodes before (curve a) and after incubation with Au NPs (curve b), BDBA (curve c) or the mixture of Au NPs and BDBA (curve d). Curve e corresponds to that of the H_2_O_2_-treated boronate-covered electrode after incubation with the mixture of Au NPs and BDBA. The final concentrations of Au NPs, BDBA and H_2_O_2_ used were 2 nM, 10 µM and 10 µM, respectively; (**B**) Effect of [peptide]/[Au NPs] ratio on *R*_et_; (**C**) Dependence of Δ*R*_et_ on H_2_O_2_ concentration (0.001, 0.01, 0.06, 0.1, 0.6, 3, 10 and 30 µM). The inset shows the linear part of the curve. The concentrations of Au NPs and BDBA used were 2 and 9 µM, respectively.

EIS has been commonly employed to monitor the molecular assembly on electrode surfaces. Herein, the sensing performances of the modified electrode were characterized by EIS. Figure 4A shows the EIS spectrum of the sensing electrode in different states. Incubating the boronate-covered electrode with the Au NP suspension caused a slight decrease in the diameter of the semicircle of the impedance spectrum (cf. curve a and curve b). The decrease suggested that Au NPs attached onto the electrode surface facilitated the electron transfer. This is attributed to the unique electrical property of Au NPs. Interestingly, incubating the boronate-covered electrode with the mixture of Au NPs and BDBA led to a much smaller semicircle portion in the impedance spectrum (curve d), but no apparent change was observed when incubating the electrode with BDBA only (curve c). The result suggested that more Au NPs were assembled on the electrode surface in the presence of BDBA. The electron transfer resistance (*R*_et_) in curve d is much lower than that in curve b, indicating that the signal could be amplified by the network of Au NPs and the BDBA-induced assembly of Au NPs in solution was facilely initiated on the electrode surface. However, incubation of the H_2_O_2_-treated boronate-covered electrode with the mixture of Au NPs and BDBA did not cause a decrease in the diameter of the semicircle (curve e), indicating that the Au NPs were incapable of assembly on the electrode surface after boronate was converted into its phenol form. Thus, H_2_O_2_ could be determined based on the change of the charge transfer resistance of the sensing electrode.

Although a high level of BDBA made the aggregation of Au NPs more powerful, a high level of BDBA in the solution could compete with the boronate on the electrode surface to bind citrate-capped Au NPs, thus reducing the sensitivity. Thus, we studied the impact of the concentration ratio of BDBA to Au NPs ([BDBA]/[Au NPs]) on the *R*_et_. It was found that *R*_et_ decreased and then increased with the increasing [BDBA]/[Au NPs] ratio (Figure 4B). The minimum value appeared at 4500:1. In the following detection assay, 4500:1 was chosen as the optimal concentration ratio. The H_2_O_2_ quantitative detection was performed by monitoring the change of *R*_et_ (Δ*R*_et_). It was observed that Δ*R*_et_ increased with the increase of the H_2_O_2_ concentration ([H_2_O_2_]). The value is proportional to [H_2_O_2_] in the range of 1 nM~0.6 µM. The regression equation is Δ*R*_et_ = 3731[H_2_O_2_] (µM) + 41, *R* = 0.997. Thus, the Au NPs–based colorimetric assay was converted into an electrochemical analysis with improved sensitivity.

### 2.4. Electrochemical Detection of H_2_O_2_ with Ag NPs as the Redox Reporters

**Figure 5:**
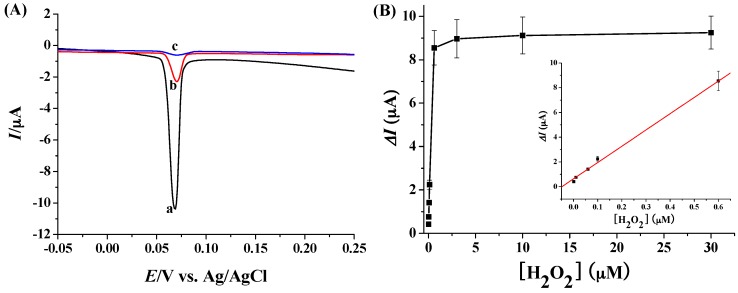
(**A**) LSV responses of the boronate-covered electrodes after incubation with the mixture of Ag NPs and BDBA (curve a) or Ag NPs only (curve b). Curve c corresponds to that of H_2_O_2_-treated boronate-covered electrode after incubation with the mixture of Ag NPs and BDBA. The final concentrations of Ag NPs, BDBA and H_2_O_2_ used were 2 nM, 8 µM and 3 µM, respectively; (**B**) Dependence of Δ*I* on H_2_O_2_ concentration (0.001, 0.01, 0.06, 0.1, 0.6, 3, 10 and 30 µM). The inset shows the linear part of the curve.

Ag NPs have been widely used as the electrochemical elements for signal outputs based on their solid-state Ag/AgCl reaction [41,42]. Particularly, the network of Ag NPs showed amplified LSV signals [13,18]. When Ag NPs were used instead of Au NPs in the proposed method, we found that the electrochemical signal could be measured by LSV based on the solid-state Ag/AgCl reaction from Ag NPs. As shown in Figure 5A, the electrochemical response of the boronate-covered electrode after incubation with Ag NPs and BDBA revealed a reduced peak at around 70 mV (curve a). The peak current was much higher than that after incubation with Ag NPs only (curve b), demonstrating that the signal was amplified by the network of Ag NPs. Expectedly, the H_2_O_2_-treated sensing electrode showed no significant LSV peak after incubation with the mixture of Ag NPs and BDBA (curve c). The result further confirmed that the assembly of Ag NPs on the electrode surface is dependent upon the formation of boronate ester bonds. We also found that the optimal concentration ratio of BDBA to Ag NPs was 4000:1. Under the optimized ratio, the LSV responses for the determination of various concentrations of H_2_O_2_ were collected. The quantitative assay was measured based on the LSV current change (Δ*I*). It was observed that Δ*I* increased with the increase of the H_2_O_2_ concentration ([H_2_O_2_]) in a linear range of 1 nM ~ 0.6 µM (Figure 5B). The regression equation is Δ*I* = 13.3[H_2_O_2_] (µM) + 0.6, *R* = 0.998.

## 3. Materials and Methods

### 3.1. Reagents and Materials

The 6-mercaptohexanoic acid (MHA), 3-aminobenzeneboronic acid (ABA), BDBA, *N*-hydroxysulfosuccinimide (NHS), 1-ethyl-3-[3-dimethylaminopropyl]carbodiimide (EDC) hydrochloride, trisodium citrate, sodium borohydride, ethanolamine, KH_2_PO_4_, and K_2_HPO_4_ were obtained from Sigma-Aldrich (Shanghai, China). All other chemicals were of analytical grade and obtained from Beijing Chemical Reagent Co. Ltd. (Beijing, China). BDBA and H_2_O_2_ were diluted with a phosphate-buffered saline solution (PBS buffer, 5 mM, pH 8.2) before use. All solutions were prepared with deionized water purified by using a Millipore system (Simplicity Plus, Millipore Corp., Billerica, MA, USA). The citrate-stabilized Au NPs with a size of 13 nm were synthesized with a trisodium citrate reduction method. The concentration of Au NPs was calculated from the UV-vis absorption spectrum by using an extinction coefficient of 2.7 × 10^8^ mol^−1^·cm^−1^ at 520 nm. Ag NPs were prepared by the chemical reduction of AgNO_3_ using sodium borohydride and trisodium citrate as the reducing reagent and the stabilizer, respectively [13]. The Ag NPs concentration was calculated based on the Ag NPs size and the Ag^+^ concentration.

### 3.2. Instruments

The UV-vis spectra were measured by a Cary 60 spectrophotometer using a 1 cm quartz spectrophotometer cell. The electrochemical measurements were conducted on a CHI 660E (CH Instruments, Shanghai, China) electrochemical workstation. The auxiliary and reference electrodes were a platinum wire and an electrode of Ag/AgCl, respectively. The distribution images of Au NPs were recorded by an FEI Tecnai G2 T20 transmission electron microscopy (TEM). The hydrodynamic diameter of the Ag NPs was measured by a Nano ZS laser scattering particles size analyzer (Malvern Instruments Ltd., Malvern, Worcestershire, UK). The photograph images were taken by a Sony Cyber-Shot digital camera (Sony Corp., Tokyo, Japan).

### 3.3. Colorimetric Detection of H_2_O_2_

To investigate the BDBA-induced Au NPs aggregation, 400 µL of Au NPs suspensions were added to 600 µL of different concentrations of BDBA solutions. After incubation for 10 min, the UV-vis absorption spectra were collected on the spectrophotometer. For the colorimetric assay of H_2_O_2_, 300 μL of H_2_O_2_ solution at a given concentration was first incubated with 300 μL of BDBA solution at ambient temperature for 1 min. Then, 400 μL of citrate-capped Au NPs suspension was added into the mixed solution. After incubation for 10 min, the UV-vis absorption spectra were recorded.

### 3.4. Electrochemical Detection of H_2_O_2_

The self-assembled monolayers (SAMs) with medium length (e.g., six carbons) can avoid non-specific absorption and allow for electron transfer. For the preparation of boronic acid-covered electrode, the cleaned polycrystalline gold disk electrode with a diameter of 2 mm) was first immersed in an ethanol solution of 1 mM MHA in the darkness for 12 h. After washed thoroughly with ethanol and water, the electrode was performed by cross-linking ABA molecules onto the carboxy-terminal SAMs surface through the EDC/NHS-mediated amine coupling reaction [43,44,45]. In brief, the MHA-covered electrode was incubated with a mixed solution comprising of 0.4 M EDC and 0.2 M NHS for 15 min, washed with water and soaked in a ABA solution (0.5 mM) for 4 h. To block the unreacted EDC/NHS-activated carboxy groups, the modified electrode was then soaked in a 1 mM ethanolamine solution for 10 min. After formation of boronic acid-modified SAMs on the electrode surface, the sensing electrode was immersed in a H_2_O_2_ solution for 1 min. After rinsed with 10 mM hydrochloric acid and water, the sensing electrode was exposed to 40 µL of Au NPs suspension in a homemade plastic cell, followed by addition of 60 µL of BDBA to incubation for 15 min. After washed with water again, the electrode was placed in a mixture of 10 mM [Fe(CN)_6_]^3−^/^4−^ (1:1) and 0.5 M KCl for impedance measurement. The potential was set at 0.245 V and the frequency ranging from 0.01 to 500 kHz. For the LSV experiments, Ag NPs were used instead of Au NPs and the electrochemical signal was collected in a 1 M KCl solution. Other experimental conditions and detection procedures were the same as those of the Au NP–based sensing strategy.

## 4. Conclusions

In this work, a metal NP–based colorimetric assay was converted into sensitive electrochemical analysis for H_2_O_2_ detection with a simple manipulation principle and an easy detection procedure. The method is based on two facts: (1) BDBA could induce the assembly of citrate-capped Au/Ag NPs via the boronate-citrate interaction; and (2) the boronate group could be removed by H_2_O_2_ to yield its corresponding phenol form, thus preventing the assembly of Au/Ag NPs. Since H_2_O_2_ is an enzymatic product, the sensing electrode could be used to measure H_2_O_2_ levels in laboratory studies and to design novel chem/bio-sensors. We also envision that the method would be expanded to quantify the levels of exogenous and endogenous H_2_O_2_ in living cells by using boronate-modified nanometer-sized electrodes.

## References

[1] Saha K., Agasti S.S., Kim C., Li X., Rotello V.M. (2012). Gold nanoparticles in chemical and biological sensing. Chem. Rev..

[2] Ronkainen N.J., Okon S.L. (2014). Nanomaterial-based electrochemical immunosensors for clinically significant biomarkers. Materials.

[3] Hayat A., Catanante G., Marty J.L. (2014). Current trends in nanomaterial-based amperometric biosensors. Sensors.

[4] Wilson R. (2008). The use of gold nanoparticles in diagnostics and detection. Chem. Soc. Rev..

[5] Sapsford K.E., Algar W.R., Berti L., Gemmill K.B., Casey B.J., Oh E., Stewart M.H., Medintz I.L. (2006). Functionalizing nanoparticles with biological molecules: Developing chemistries that facilitate nanotechnology. Chem. Rev..

[6] Khalil I., Julkapli N.M., Yehye W.A., Basirun W.J., Bhargava S.K. (2016). Graphene-gold nanoparticles hybrid-synthesis, functionalization, and application in a electrochemical and surface-enhanced Raman scattering biosensor. Materials.

[7] Bülbül G., Hayat A., Andreescu S. (2015). Portable nanoparticle-based sensors for food safety assessment. Sensors.

[8] Elghanian R., Storhoff J.J., Mucic R.C., Letsinger R.L., Mirkin C.A. (1997). Selective colorimetric detection of polynucleotides based on the distance-dependent optical properties of gold nanoparticles. Science.

[9] Liu L., Zhao F., Ma F., Zhang L., Yang S., Xia N. (2013). Electrochemical detection of β-amyloid peptides on electrode covered with *N*-terminus-specific antibody based on electrocatalytic O_2_ reduction by Aβ(1–16)-heme-modified gold nanoparticles. Biosens. Bioelectron..

[10] Liu P.D., Jin H.Z., Guo Z.R., Ma J., Zhao J., Li D.D., Wu H., Gu N. (2016). Silver nanoparticles outperform gold nanoparticles in radiosensitizing U251 cells in vitro and in an intracranial mouse model of glioma. Int. J. Nanomed..

[11] McFarland A.D., Van Duyne R.P. (2003). Single silver nanoparticles as real-time optical sensors with zeptomole sensitivity. Nano Lett..

[12] Nam J.-M., Thaxton C.S., Mirkin C.A. (2003). Nanoparticle-based bio-bar codes for the ultrasensitive detection of proteins. Science.

[13] Xia N., Wang X., Zhou B., Wu Y., Mao W., Liu L. (2016). Electrochemical detection of amyloid-β oligomers based on the signal amplification of a network of silver nanoparticles. ACS Appl. Mater. Interfaces.

[14] Xia N., Zhou B., Huang N., Jiang M., Zhang J., Liu L. (2016). Visual and fluorescent assays for selective detection of beta-amyloid oligomers based on the inner filter effect of gold nanoparticles on the fluorescence of CdTe quantum dots. Biosens. Bioelectron..

[15] Chang H.C., Ho J.A.A. (2015). Gold nanocluster-assisted fluorescent detection for hydrogen peroxide and cholesterol based on the inner filter effect of gold nanoparticles. Anal. Chem..

[16] Xu J., Yu H., Hu Y., Chen M.Z., Shao S.J. (2016). A gold nanoparticle-based fluorescence sensor for high sensitive and selective detection of thiols in living cells. Biosens. Bioelectron..

[17] Xia N., Wang X., Wang X., Zhou B. (2016). Gold nanoparticle-based colorimetric and electrochemical methods for dipeptidyl peptidase-IV activity assay and inhibitor screening. Materials.

[18] Wei T., Dong T., Wang Z., Bao J., Tu W., Dai Z. (2015). Aggregation of individual sensing units for signal accumulation: Conversion of liquid-phase colorimetric assay into enhanced surface-tethered electrochemical analysis. J. Am. Chem. Soc..

[19] Sobotta M.C., Liou W., Stocker S., Talwar D., Oehler M., Ruppert T., Scharf A.N., Dick T.P. (2015). Peroxiredoxin-2 and STAT3 form a redox relay for H_2_O_2_ signaling. Nat. Chem. Biol..

[20] Lippert A.R., Van de Bittner G.C., Chang C. (2011). Boronate oxidation as a bioorthogonal reaction approach for studying the chemistry of hydrogen peroxide in living systems. Acc. Chem. Res..

[21] Noh J., Kwon B., Han E., Park M., Yang W., Cho W., Yoo W., Khang G., Lee D. (2015). Amplification of oxidative stress by a dual stimuli-responsive hybrid drug enhances cancer cell death. Nat. Commun..

[22] Li R., Liu X., Qiu W., Zhang M. (2016). In vivo monitoring of H_2_O_2_ with polydopamine and prussian blue-coated microelectrode. Anal. Chem..

[23] Gu X., Wang H., Schultz Z.D., Camden J.P. (2016). Sensing glucose in urine and serum and hydrogen peroxide in living cells by use of a novel boronate nanoprobe based on surface-enhanced Raman spectroscopy. Anal. Chem..

[24] Wang Y., Hasebe Y. (2014). Carbon felt-based bioelectrocatalytic flow-through detectors: 2,6-Dichlorophenol indophenol and peroxidase coadsorbed carbon-felt for flow-amperometric determination of hydrogen peroxide. Materials.

[25] De Gracia Lux C., Joshi-Barr S., Nguyen T., Mahmoud E., Schopf E., Fomina N., Almutairi A. (2012). Biocompatible polymeric nanoparticles degrade and release cargo in response to biologically relevant levels of hydrogen peroxide. J. Am. Chem. Soc..

[26] Michalski R., Zielonka J., Gapys E., Marcinek A., Joseph J., Kalyanaraman B. (2014). Real-time measurements of amino acid and protein hydroperoxides using coumarin boronic acid. J. Biol. Chem..

[27] Van de Bittner G.C., Dubikovskaya E.A., Bertozzi C.R., Chang C.J. (2010). In vivo imaging of hydrogen peroxide production in a murine tumor model with a chemoselective bioluminescent reporter. Proc. Natl. Acad. Sci. USA.

[28] Webb K.S., Levy D. (1995). A facile oxidation of boronic acids and boronic esters. Tetrahedron Lett..

[29] Zhang H., Ren T., Ji Y., Han L., Wu Y., Song H., Bai L., Ba X. (2015). Selective modification of halloysite nanotubes with 1-pyrenylboronic acid: A novel fluorescence probe with highly selective and sensitive response to hyperoxide. ACS Appl. Mater. Interfaces.

[30] Miller E.W., Albers A.E., Pralle A., Isacoff E.Y., Chang C.J. (2005). Boronate-based fluorescent probes for imaging cellular hydrogen peroxide. J. Am. Chem. Soc..

[31] Manimala J.C., Wiskur S.L., Ellington A.D., Anslyn E.V. (2004). Tuning the specificity of a synthetic receptor using a selected nucleic acid receptor. J. Am. Chem. Soc..

[32] Bosch L.I., Fyles T.M., James T.D. (2004). Binary and ternary phenylboronic acid complexes with saccharides and Lewis bases. Tetrahedron.

[33] Wiskur S.L., Lavigne J.J., Metzger A., Tobey S.L., Lynch V., Anslyn E.V. (2004). Thermodynamic analysis of receptors based on guanidinium/boronic acid groups for the complexation of carboxylates, a-hydroxycarboxylates, and diols: Driving force for binding and cooperativity. Chem. Eur. J..

[34] Yang Y.-C., Tseng W.-L. (2016). 1,4-Benzenediboronic-acid-induced aggregation of gold nanoparticles: Application to hydrogen peroxide detection and biotin-avidin-mediated immunoassay with naked-eye detection. Anal. Chem..

[35] Miao P., Wang B., Han K., Tang Y. (2014). Electrochemical impedance spectroscopy study of proteolysis using unmodified gold nanoparticles. Electrochem. Commun..

[36] Yang Y., Li C., Yin L., Liu M., Wang Z., Shu Y., Li G. (2014). Enhanced charge transfer by gold nanoparticle at DNA modified electrode and its application to label-free DNA detection. ACS Appl. Mater. Interfaces.

[37] Zhao J., Zhu X., Li T., Li G. (2008). Self-assembled multilayer of gold nanoparticles for amplified electrochemical detection of cytochrome c. Analyst.

[38] Anzai J. (2016). Recent progress in electrochemical biosensors based on phenylboronic acid and derivatives. Mater. Sci. Eng. C-Mater..

[39] Egawa Y., Miki R., Seki T. (2014). Colorimetric sugar sensing using boronic acid-substituted azobenzenes. Materials.

[40] Liu L., Xia N., Liu H.P., Kang X.J., Liu X.S., Xue C., He X.L. (2014). Highly sensitive and label-free electrochemical detection of microRNAs based on triple signal amplification of multifunctional gold nanoparticles, enzymes and redox-cycling reaction. Biosens. Bioelectron..

[41] Lin D., Wu J., Wang M., Yan F., Ju H. (2012). Triple signal amplification of graphene film, polybead carried gold nanoparticles as tracing tag and silver deposition for ultrasensitive electrochemical immunosensing. Anal. Chem..

[42] Singh P., Parent K.L., Buttry D.A. (2012). Electrochemical solid-state phase transformations of silver nanoparticles. J. Am. Chem. Soc..

[43] Liu L., Deng D., Xing Y., Li S., Yuan B., Chen J., Xia N. (2013). Activity analysis of the carbodiimide-mediated amine coupling reaction on self-assembled monolayers by cyclic voltammetry. Electrochim. Acta.

[44] Liu S., Wollenberger U., Halámek J., Leupold E., Stçcklein W., Warsinke A., Scheller F.W. (2005). Affinity Interactions between phenylboronic acid-carrying self-assembled monolayers and flavin adenine dinucleotide or horseradish peroxidase. Chem. Eur. J..

[45] Yildiz H.B., Freeman R., Gill R., Willner I. (2008). Electrochemical, photoelectrochemical, and piezoelectric analysis of tyrosinase activity by functionalized nanoparticles. Anal. Chem..

